# Evaluation of Commercial Prototype Bacteriophage Intervention Designed for Reducing O157 and Non-O157 Shiga-Toxigenic *Escherichia coli* (STEC) on Beef Cattle Hide

**DOI:** 10.3390/foods7070114

**Published:** 2018-07-16

**Authors:** Tamra N. Tolen, Yicheng Xie, Thomas B. Hairgrove, Jason J. Gill, T. Matthew Taylor

**Affiliations:** Department of Animal Science, Texas A&M University, College Station, TX 77843-2471, USA; ttolen84@tamu.edu (T.N.T.); seasonxie@tamu.edu (Y.X.); tbhairgrove@tamu.edu (T.B.H.); jason.gill@tamu.edu (J.J.G.)

**Keywords:** Shiga-toxigenic *E. coli*, bacteriophages, beef safety, biocontrol, cattle hides, pre-harvest

## Abstract

Microbiological safety of beef products can be protected by application of antimicrobial interventions throughout the beef chain. This study evaluated a commercial prototype antimicrobial intervention comprised of lytic bacteriophages formulated to reduce O157 and non-O157 Shiga-toxigenic *Escherichia coli* (STEC) on beef cattle hide pieces, simulating commercial pre-harvest hide decontamination. STEC reduction in vitro by individual and cocktailed phages was determined by efficiency of plating (EOP). Following STEC inoculation onto hide pieces, the phage intervention was applied and hide pieces were analyzed to quantify reductions in STEC counts. Phage intervention treatment resulted in 0.4 to 0.7 log_10_ CFU/cm^2^ (*p* < 0.01) *E. coli* O157, O121, and O103 reduction. Conversely, *E. coli* O111 and O45 did not show any significant reduction after application of bacteriophage intervention (*p* > 0.05). Multiplicity of infection (MOI) evaluation indicated *E. coli* O157 and O121 isolates required the fewest numbers of phages per host cell to produce host lysis. STEC-attacking phages may be applied to assist in preventing STEC transmission to beef products.

## 1. Introduction

The Shiga-toxigenic *Escherichia coli* (STEC), including O157 and non-O157 serogroups, are important foodborne human pathogens, often associated with consumption of improperly prepared or undercooked beef products. The severity of disease for O157 and non-O157 STEC disease cases can vary from moderate acute symptoms to debilitating chronic sequelae, and can result in premature death [1]. In addition to post-harvest cross-contamination of beef carcasses and products with members of the O157 and non-O157 Shiga-toxigenic *Escherichia coli* (STEC), these human pathogens have been recovered from live cattle prior to animal slaughter [2,3,4]. Members of the STEC may reside as commensals in the bovine GI tract, and have been previously associated with human disease outbreaks involving consumption of cross-contaminated fresh non-intact beef products (e.g., ground beef hamburgers, mechanically tenderized beef steaks) [5,6,7,8]. Post-harvest beef carcass safety interventions, including hide wash cabinets for hide-on carcasses [9], steam vacuuming of hide-off carcasses [10,11], knife trimming [12], organic acids [13], and hot water sprays [14,15], in addition to multiple other chemical and physical interventions, have shown success in reducing numbers and/or prevalence of O157 and non-O157 STEC on beef carcasses. Nevertheless, need remains to develop and evaluate novel antimicrobial interventions, which can be applied during handling of cattle to reduce STEC on exterior surfaces of animals presented for harvest.

Lytic bacteriophages (phages) are viruses that infect and replicate within bacterial host cells, causing lysis of the host with accompanying release of new virions. Bacteriophages have been applied onto different food products and food contact surfaces to reduce bacterial pathogens [16,17,18,19]. In addition, the U.S. Department of Agriculture—Food Safety and Inspection Service (USDA-FSIS) has approved multiple bacteriophage-containing antimicrobial products for pathogen reduction on animals prior to slaughter, including the O157 STEC [20]. Phages as pre-harvest antimicrobial interventions for meat animals have been shown to reduce pathogen shedding in feces by oral delivery of phages [21,22,23], and to reduce animal hide surface counts of O157 STEC through application onto hides. Coffey et al. [24] reported a 2.0 log_10_ CFU/cm^2^ reduction in *E. coli* O157:H7 numbers on hide pieces treated with a pair of lytic phages recovered from cattle. Arthur et al. [25], however, reported no statistical difference between the numbers of O157 STEC-positive cattle hides for cattle treated with a commercial O157 STEC-infecting phage intervention versus those treated with a water wash prior to slaughter. To our knowledge, no research is available in the refereed literature detailing the utility of phages for hide decontamination of the non-O157 STEC. The objective of this study was to evaluate the capacity of a commercially produced prototype phage intervention designed to infect and lyse members of the O157 and non-O157 STEC on inoculated beef cattle hide pieces to simulate live animal STEC decontamination prior to slaughter. The null hypothesis was that phage application would not result in statistically detectable differences in STEC survivors versus the non-treated STEC-inoculated control.

## 2. Materials and Methods

### 2.1. STEC Host Range and Spot Titration of Phages

Shiga-toxigenic *E. coli* isolates, all naturally resistant to the RNA polymerase inhibitor rifampicin (100 µg/mL), belonging to serogroups O157, O45, O103, O111, and O121, O26 and O145 were revived from −80 °C cryo-storage in the Food Microbiology Laboratory culture collection (Department of Animal Science, Texas A&M University, College Station, TX, USA) (Table 1). All isolates were first revived in tryptic soy broth (TSB; Becton, Dickinson and Co., Sparks, MD, USA) by aseptically inoculating a loop of frozen culture in TSB and then incubating with agitation at 37 °C for 24 h. Following incubation, isolates were individually streaked for isolation onto surfaces of tryptic soy agar (TSA; Becton, Dickinson and Co., Franklin Lakes, NJ, USA) and incubated at 37 °C overnight. An isolated colony of each STEC isolate was then inoculated into 2.0 mL TSB and held in a roller drum at 37 °C overnight. Following incubation, 30.0 µL was aseptically pipetted into 3.0 mL sterile TSB, and incubated at 37 °C with agitation until the optical density at 550 nm (OD_550_) reached 0.5. Cultures were adjusted to OD_550_ = 0.5 with sterile TSB as needed, giving an approximate count of 10^8^ CFU/mL for each STEC isolate, and then held on ice until ready for use.

Phages were plated using the soft agar overlay method. Lawns were composed of 5.0 mL top agar (10.0 g/L Bacto tryptone, 10.0 g/L NaCl, 5.0 g/L Bacto agar) inoculated with 100.0 µL overnight host culture poured over tryptic soy agar (TSA; Becton, Dickinson and Co., Franklin Lakes, NJ, USA) bottom plates [26]. The molten agar was supplemented with 5.0 mM MgSO_4_ (Millipore Sigma, Burlington, MA, USA) and 5.0 mM CaCl_2_ (Macron Chemicals™, Avantor Performance Materials, Inc., Center Valley, PA, USA) before use. The molten agar was aliquoted into plastic tubes (5.0 mL each), placed in a heating block and allowed to temper to 50 °C. For each STEC isolate, 100.0 µL of culture (OD_550_ = 0.5) was transferred to a 5.0 mL molten agar tube and vortexed gently for 1.0 s before pouring onto a TSA plate. Plates were swirled in order to distribute the molten agar completely across the TSA surface. Plates were then set at ambient conditions for 1–5 min to allow top agar to cool and solidify.

### 2.2. Efficiency of Plating Assay for Phage Infection of STEC Hosts

A commercial prototype phage intervention was provided by Passport Food Safety Solutions, Inc. (W. Des Moines, IA, USA), both as a cocktail of STEC-infecting phages and individual constituent phages (each at ~10^8^ PFU/mL). While STEC-inoculated plates were solidifying, phages (individual or pre-mixed cocktail) were serially diluted 10^−1^ to 10^−7^. Efficiency of plating (EOP) assays were performed to determine how well individual phages or the mixed cocktail of phages infected each STEC serogroup by published methods [27,28]. Evaluation of STEC isolate and phage spot-tests was completed to determine EOP outcomes for each STEC host/phage pairing (individual phages or phage cocktail). The spot-test reports the number of phage infecting a host bacterium per mL of applied phage suspension, and can be impacted by variations in host susceptibility between host isolates [27,28]. The *E. coli* O157:H7 isolate was used as the reference STEC for EOP assignment for experiments using the cocktailed phage intervention. In experiments applying individual phages against STEC isolates, the STEC isolate bearing the highest susceptibility (highest count of phage progeny resulting from infection) was assigned as the reference STEC for that particular phage. EOP assays were replicated identically three times, with one sample per replicate (*N =* 3) and mean EOP values calculated from like samples. Following the conclusion of EOP assays, the five STEC isolates comprising a range of high (>0.5), moderate (0.1–0.5), and low (<0.1) EOP were subsequently selected to proceed further through in vitro STEC growth inhibition and high application studies. This was done to provide a compilation of STEC bearing the highest to lowest infection susceptibility to phage intervention application, in an effort to improve the accuracy of estimation of the intervention’s efficacy against STEC on hides.

### 2.3. In Vitro Growth Inhibition of STEC by Phages

STEC isolates were prepared for growth inhibition assays using the same method described above (Section 2.1). Following adjustment of culture-containing TSB to OD_550_ = 0.5, cultures were placed on ice until ready for further use. A target inoculum of approximately 10^5^ CFU/mL of each STEC isolate was prepared by adding 0.01 mL of a culture to 10 mL sterile TSB. Final STEC counts were determined by preparation of serial dilutions in 0.1% peptone (*w*/*v*) diluent (Becton, Dickinson and Co., Franklin Lakes, NJ, USA) and spreading on surfaces of TSA supplemented with 100.0 µg/mL rifampicin (TSAR). Inoculated plates were incubated for 24 h at 35 °C prior to colony enumeration.

To evaluate the effects of the phage cocktail on STEC isolates’ growth in liquid culture, 160.0 µL of TSB (not inoculated) was loaded into five consecutive wells in a single row on a sterile 96-well polystyrene microplate (Corning Inc., Tewksbury, MA, USA) [27]. Twenty microliters each of individual STEC isolate (see Table 1 for STEC isolates identification), each serially diluted to provide a target inoculum of 10^6^ CFU/mL, were then added to test wells. To the first well in each row, 20 µL of undiluted phage cocktail was added to the STEC isolate-containing well and mixed by repeated pipetting. The phage intervention was serially diluted ten-fold by transfer of 20 µL each to subsequent wells, producing four 10-fold serial dilutions of phages, ranging from 10^−1^ to 10^−4^ from the original phage product. The fifth well of the plate remained untreated with phages and served as a STEC-positive control. The phage cocktail was also inoculated into TSB-only wells (negative controls) in a similar fashion to STEC-inoculated wells to verify no cross-contamination of phages with phage-immune hosts or other microbes and provide for a baseline adjustment to the gathered data. The prepared plate was covered and held in a shaking, temperature-controlled plate reader (Tecan 10M Spark, Tecan U.S., Inc., Morrisville, NC, USA) at 37 °C for 12 h with shaking. Absorbance at 550 nm was read every 30 min to determine host inhibition or growth. After the 12 h time period, baseline adjustment was completed by taking OD_550_ readings of phage/TSB-only wells and subtracting from the value at each time point for each STEC isolate tested. Baseline-adjusted values were then compared for phage-treated STEC at each dilution of phages against the positive control for a specific STEC isolate.

### 2.4. Application of Decontamination Treatments to STEC-Inoculated Cattle Hide Pieces and Determination of Phage: Host Multiplicity of Infection (MOI)

STEC isolates were prepared for inoculation onto cattle hide pieces to determine the capacity of the prototype phage intervention for reducing O157 and non-O157 STEC on cattle hides. Hides were collected from inspected cattle slaughter establishments within a 56.3 km radius surrounding College Station, TX, following pulling, but before any antimicrobial treatments were applied. Hides were stored at 5 °C and obtained from the establishment no more than 24 h post-slaughter. Previous research has indicated no significant change in the microbiological profile, hair characteristics, as well as little shrinkage of tissue, during post-hide removal refrigerated storage [29]. A total of three hides were purchased per visit, from which hide pieces were taken from the round toward the dorsal and rear end of the hide using a hide cutting press. The round was chosen during preliminary trials wherein hide pieces taken from the round retained their shape and did not significantly shrink following overnight refrigeration in contrast to pieces taken from the flank or belly. Pieces taken from a single animal hide were packaged and separated from like pieces from other hides to preserve against hide-to-hide cross-contamination, and to allow pieces to be used as independent samples. Hide pieces were immediately returned to the Food Microbiology Laboratory where they were washed with distilled water to remove loose debris, similar to previous studies detailing phage decontamination of cattle hides [24]. The pieces were placed on sanitary plastic racks hair side up and angled to facilitate draining of excess water. Washed hides were then maintained for 2 h at room temperature to facilitate drying. Pieces from like hides were then vacuum packaged (Model X200, Koch Supplies, Inc., Kansas City, MO, USA; vacuum pressure: −95,000 N/m^2^) with paper towels and refrigerated at 5 °C.

Upon initiating experimentation, hide pieces were taken out of cold storage and allowed to warm for 2 h at 37 °C before opening vacuum pouches for subsequent inoculation and testing. Three circular pieces were taken from a single hide for each treatment group and surface area for each piece was recorded. Overnight cultures of STEC isolates were diluted in 0.1% (*w*/*v*) peptone to achieve a 10^7^ CFU/mL inoculum and loaded into a hand-trigger spray bottle. Individual hide pieces were aseptically placed hair side up in Ziploc^®^ gallon-sized stand-and-fill bags and spray-inoculated with the inoculum, delivering a total volume of 2.0 mL inoculum onto the surface of the hide piece. The inoculum was discharged from the spray bottle three to five centimeters from the hide surface while residing inside the Ziploc^®^ bag. The pieces were then allowed 30 min for STEC inoculum to attach to hide surfaces at 25 °C prior to treatment.

After the attachment period was completed, inoculated hide pieces were aseptically transferred to new Ziploc^®^ gallon-sized stand-and-fill bags containing wet paper towels set beneath a perforated plastic base to prevent excess hide dehydration. Experimental treatments were: (1) STEC-inoculated non-treated group (Control); (2) STEC-inoculated, sterile distilled water (25 °C; designed to determine any STEC removal via washing effects of intervention spraying); and (3) STEC-inoculated with 10^8^ PFU/mL phage intervention-treated. Three hide piece samples were left non-inoculated and untreated to detect the presence of any background Rif^R^ organisms. Hide pieces were sprayed with either sterile water or the phage intervention delivering a total of 2.0 mL of treatment to a hide piece. Ziploc^®^ bags were sealed and placed in a 37 °C incubator for a 1.0 h dwell period. Immediately afterwards, pieces were aseptically transferred to filter stomacher bags and stomached in 100 mL chilled phosphate-buffered saline (PBS; Thermo-Fisher Scientific, Inc., Waltham, MA, USA) for 2.0 min. Ten mL stomached sample was extracted and centrifuged for 10 min at 8500× *g* at 4 °C to separate non-adsorbed phages from STEC cells (intervention neutralization). Following centrifugation, resulting supernatant was discarded and the pellet suspended in 10 mL PBS to wash STEC cells from non-adsorbed phages. The sample was then serially diluted in PBS and plated onto TSAR supplemented with 0.1% cycloheximide (Millipore Sigma, Burlington, MA, USA) to suppress fungi growth. The plates were incubated for 24 h at 37 °C, after which STEC colonies were counted and recorded. Counts were then adjusted to report STEC survival per cm^2^ for each treatment, and therereafter log_10_ transformed. Data were generated from three identically completed experimental replicates, each containing three identically handled hide samples per STEC isolate and intervention pairing (*N* = 9).

Following completion of studies on hide decontamination, the multiplicity of infection (MOI; the ratio of phages to bacterium applied to the system) was retrospectively calculated to further evaluate the performance of the phage intervention for hide decontamination. To determine a STEC isolate/phage-specific MOI, surface-retained STEC counts (CFU/cm^2^) were transformed to total CFU by multiplying by the corresponding hide surface area. The ratio of hide surface-retained STEC to the count of STEC initially inoculated onto hides was determined as a proxy for estimating the numbers of phages retained on hides following application. Estimated retained phage was then divided by the surface area of the hide piece to yield an applied phage count (PFU/cm^2^). The resulting PFU/cm^2^ was then multiplied by the EOP of the phage on the STEC isolate in question, to produce a phage titer. This was then divided by the CFU/cm^2^ to give an applied MOI.

### 2.5. Statistical Analysis of Data

Mean STEC survivor counts calculated from hide piece decontamination experiments (log_10_ CFU/cm^2^) were analyzed by analysis of variance (ANOVA) to determine differences between STEC survivor counts as a function of antimicrobial treatment (control, phage-treated, water-washed), STEC isolate, and the interaction of these main effects. Statistically significant differences between least squares means were separated via Tukey’s Honestly Significant Differences (HSD) means separation test at *p* = 0.05.

## 3. Results

### 3.1. Efficiency of Plating of Individual and Cocktailed Phages against STEC Isolates

Spot testing and EOP assay results using the individual phages, and the mixed phage intervention product, are given in Table 1. Applied individually, phage A infected the O111 isolate only, while other phages infected multiple STEC isolates belonging to differing serogroups. When applied as a cocktail, phages were equally effective at infection of O157:H7 and O103:H2 STEC isolates, demonstrating greater infection efficiency against STEC O111:H- but reduced infection efficiency against other STEC isolates. Data analysis indicated *E. coli* O26 and O145 isolates exhibited moderate and low EOP with respect to phage cocktail infection, respectively, and were subsequently discontinued from further study and testing. The five STEC comprising the entire range of EOP (Table 1) were then submitted for subsequent testing and hide application study.

### 3.2. STEC Growth In Vitro Challenged with Serially Diluted Phages

**Figure 1:**
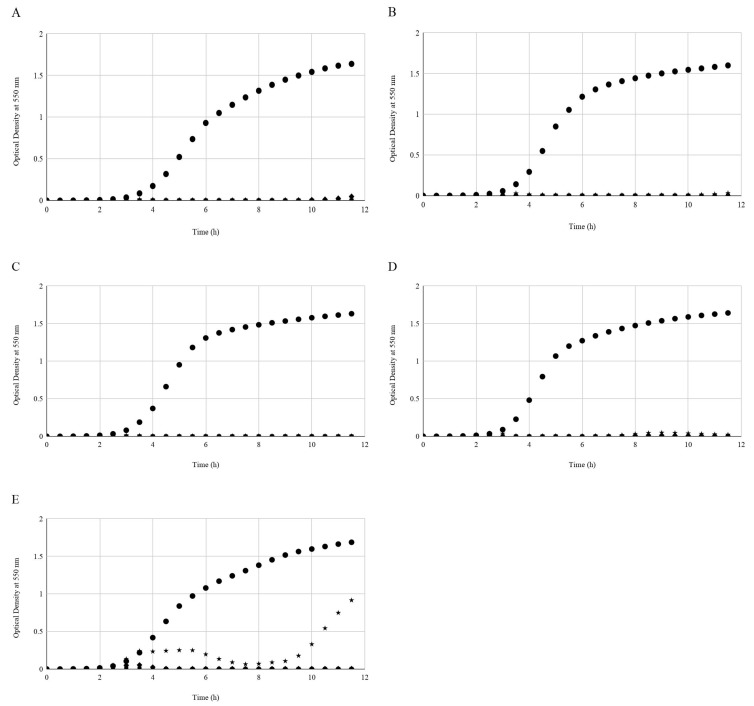
Growth or inhibition of Shiga-toxigenic *E. coli* (STEC) isolates in vitro as a function of phage dilution. Symbols depict mean optical density at 550 nm (OD_550_) from triplicate identical replicates (*N* = 3) for STEC isolates grown in the absence (Control; ●), phages diluted 10^−1^ from commercial phage intervention (▲), phages diluted 10^−2^ (∎) phages diluted 10^−3^ (◆), or phages diluted 10^−4^ (⋆). Panels relate the growth of *E. coli* O157:H7 (**A**), O45:H2 (**B**), O103:H2 (**C**), O111:H- (**D**), or O121:H19 (**E**).

For all isolates the STEC-inoculated non-treated wells showed uninhibited growth of the host isolate (Figure 1). Phages inhibited the growth of STEC isolates over the 12 h period where OD_550_ values remained unchanged for the 10^−1^-diluted phage treatment. The trend observed for all phage dilutions (10^−1^ to 10^−4^) was that the OD_550_ values of STEC isolates never rose higher than 0.2 units over the 12 h period. The only exception was with the *E. coli* O121 host isolate, which showed weak growth in the presence of the phages at dilution 10^−4^. There was initial inhibition of the isolate between 3.0 and 5.5 h incubation; thereafter, OD_550_ values decreased until the 9.0 h point, after which values slowly increased (Figure 1).

### 3.3. STEC Reduction on Cattle Hide Pieces by Differing Antimicrobial Treatments and Estimation of Numbers of Phages Required to Infect Host Cells by the Multiplicity of Infection (MOI) Determination

Hide pieces measuring an average surface area of 67.2 ± 6.0 cm^2^ were individually inoculated with 7.7 ± 0.2 log_10_ CFU/mL *E. coli* O157:H7, 7.1 ± 0.6 log_10_ CFU/mL *E. coli* O45:H2, 7.6 ± 0.3 log_10_ CFU/mL *E. coli* O103:H2, 7.7 ± 0.1 log_10_ CFU/mL *E. coli* O111:H-, or 7.0 ± 0.1 log_10_ CFU/mL *E. coli* O121:H19 for decontamination treatment. Resulting mean numbers for each STEC isolate retained on the surfaces of hide pieces following inoculation and attachment are presented in Table 2. The interaction of STEC isolate by antimicrobial treatment was highly significant for describing STEC survival on hide pieces (*p* < 0.01) (Table 2). Counts of *E. coli* O157:H7, O103:H2 or O121:H19 differed between treatments; STEC isolates were reduced by phage application versus the untreated control and water-sprayed hide pieces (Table 2). The O157:H7 isolate mean survivor count for phage-treated hide pieces, 5.6 ± 0.2 log_10_ CFU/cm^2^, was 0.5 log_10_ CFU/cm^2^ lower than on non-treated hide samples. There was no difference between STEC O157 counts on water-sprayed hide pieces (6.1 ± 0.2 log_10_ CFU/cm^2^) when compared to untreated control hide pieces. A reduction was achieved with the *E. coli* O103:H2 isolate by phage application, wherein host counts on non-treated hide pieces were 0.7 log_10_ CFU/cm^2^ higher than on phage-treated hide samples (5.2 ± 0.4 log_10_ CFU/cm^2^) (*p* < 0.05). Counts of the host on control hide pieces, nonetheless, did not differ from those on water-sprayed hide pieces (6.0 ± 0.2 log_10_ CFU/cm^2^) (Table 2). Survivors of *E. coli* O121:H19-inoculated hide pieces receiving no treatment (5.1 ± 0.2 log_10_ CFU/cm^2^) were statistically higher than phage-treated samples (4.7 ± 0.3 log_10_ CFU/cm^2^). Conversely, there were no differences detected when comparing *E. coli* O121:H19 counts on non-treated hide pieces to those from pieces treated with sterile water. Finally, hide sample pieces inoculated with *E. coli* O45:H2 and O111:H- bore no differences in mean survivor counts between treatments (Table 2).

Higher MOI values indicate a higher estimated count of phages per bacterium applied within the test system. An MOI of 10 indicates 10 phage PFU produced per *E. coli* CFU were applied. The effective applied MOIs ranged from 2.2 to 47.3, reflecting the variation in plating efficiency of the phages against different *E. coli* strains. Accordingly, STEC isolates supporting lower EOPs also had lower effective MOIs in the hide treatment system (*E. coli* O121, O157, and O103 isolates), as a uniform amount of phage was applied in all cases (Table 2).

## 4. Discussion

The four phages contained in the prototype intervention were not equally infectious to all STEC isolates tested (Table 1). The *E. coli* O103:H2 isolate was susceptible to infection by three of four phages in the prototype intervention, whereas the O157:H7 and O111:H- isolates were sensitive to only two phages each. *E. coli* O45:H2 and O121:H19 isolates were infected by only one phage in the intervention. These were also the only two isolates sensitive to phage C in the cocktailed intervention product, which raises the possibility that phage C possesses greater bactericidal activity in situ than other phages included. *E. coli* O157:H7 and O103:H2 isolates were sensitive to phage B; the *E. coli* O45:H2 isolate was weakly susceptible to phage B but was not reduced by application of the intervention (cocktail of phages). The *E. coli* O111:H- strain used in this study was susceptible to phages A and D but was not reduced by cocktailed phages on hides. This perhaps indicates these phages did not sufficiently contact the host on the hide surface to effect pathogen reduction, possibly due to the complexity of the hide surface, irregularities in hair length, or incomplete mud or feces removal [25]. These findings highlight one of the challenges in formulating a phage-containing antimicrobial intervention capable of acting on complex surfaces against a large range of targeted hosts. While the intervention may contain phages capable of significant in vitro host infection/lysis, similar infection capacities may not be observed during intended industrial usage. Measurements beyond phage infection efficiency via methods such as EOP and/or MOI are likely required to develop, deploy, and maintain effective phage interventions. STEC isolates used in the current study were harvested from differing sources, including multiple isolates recovered from human stool samples from sporadic or outbreak-associated disease cases, and from beef products [30]. While the use of only one isolate per serogroup limits the opportunity for generalization of experimentally-obtained STEC reductions to the broader STEC ecology in beef production, data presented do indicate the usefulness of STEC-infecting phages in combination with other food safety interventions applied within the beef chain.

Phage/host EOP or applied MOI did not appear to be strong predictors of phage efficacy on hide pieces. While reductions were observed for *E. coli* O157 and O103 isolates treated by phages, *E. coli* O111 counts were not reduced by treatment despite having the highest in vitro EOP and applied MOI (Table 1 and Table 2). *E. coli* O121 was statistically reduced (*p* < 0.01) following phage treatment, even though it had the lowest EOP (0.01) and applied MOI (2.2). Based on phage MOIs, the maximum theoretical reductions in bacterial loads would be approximately 1.0 log_10_-cycle for *E. coli* O121, and greater than 4.0 log_10_-cycles for other STEC isolates, assuming every applied phage encountered a bacterial host cell in a randomly-assorted mode with a Poisson distribution [26]. This was not observed in the current study, suggesting interference of phage adsorption to hosts in the heterogeneous environment of the cattle hide. The authors speculate that impedance of phage diffusion, bacterial residence in protected sites, adsorption of phages to hair follicles, or limitations in availability of free water and divalent cations may also have played roles in observed phage efficacy in this study. More study is needed to determine if phage application to cattle can prevent pathogen contamination from treated hides onto carcasses, thus reducing the transfer of organisms to resulting food products.

The purpose of this research was to determine if the application of a multi-phage prototype antimicrobial intervention applied to cattle hide pieces would reduce inoculated O157 and non-O157 STEC. The STEC have been associated with foodborne disease outbreaks where the consumption of contaminated beef was identified as a risk factor for human disease [31,32,33,34,35]. Lytic phages that can be applied to cattle pre-harvest to decontaminate hides or other external animal surfaces (e.g., hooves) represent a useful intervention technology to add further pathogen reduction to those achieved by post-harvest food safety interventions. In the current study, *E. coli* isolates belonging to differing serogroups of O157 and non-O157 STEC were reduced (*p* < 0.05) by phage application, though not to the extent reported in other studies evaluating the efficacy of anti-*E. coli* O157:H7 phages on cattle hide pieces [24]. Other research investigating electrolyzed water at near neutral pH, useful for live cattle treatment to reduce STEC without animal welfare compromise, reported similar reduction in *E. coli* O157:H7 numbers to those reductions reported here (0.7 log_10_ CFU/cm^2^) [36]. Nonetheless, Elramady et al. [37] reported the combination of 1.0% lactic acid with 1.0% sodium dodecyl sulfate reduced *E. coli* O157:H7 on cattle hides by 4.6 log_10_ CFU/cm^2^, a substantially greater reduction than those observed in the current study for O157 and non-O157 STEC members. Additionally, the current methods of phage characterization provide only limited utility in predicting interactions of phages and targeted hosts in complex environments such as the hides of beef cattle prior to harvest.

## Figures and Tables

**Table 1:** Phage titer and efficiency of plating characterization of Shiga-toxigenic *E. coli* (STEC) by phages.

STEC Isolate ^1^	Sero-ID	Phage Cocktail Titer (log_10_ PFU/mL) ^2^	Phage Efficiency of Plating (EOP)				
			Phage A	Phage B	Phage C	Phage D	Phage Cocktail ^4^
USDA-FSIS 380-94	O157:H7	8.6 ± 0.1	-	0.19 ^3^	1.0	+	1.0
CDC 96-3258	O45:H2	8.3 ± 0.1	+	0.08	-	+	0.5
CDC 90-3128	O103:H2	8.4 ± 0.5	+	1.0	0.42	0.25	1.0
JB1-95	O111:H-	9.1 ± 0.1	1.0	-	-	0.29	3.3
CDC 97-3068	O121:H19	6.7 ± 0.1	-	-	-	0.08	0.01

^1^ STEC isolates provided by J.B. Luchansky (USDA-Agricultural Research Service, Wyndmoor, PA, USA). ^2^ Values depict mean ± one standard deviation (*N* = 3) of phages plated for corresponding STEC isolate. ^3^ EOP calculated as phage titer on test STEC isolate divided by phage titer on the *E. coli* O157:H7 isolate for phages A, B, and C. EOP for phage D was normalized to phage titer on *E. coli* O27:H11 isolate H30 (data not shown). -: no observed plaques or clearing zones formed at any phage dilution; +: confluent spot formation at 10^−1^–10^−2^ phage dilutions but no isolated plaques observed. ^4^ EOP calculated as observed phage titer on the STEC isolate divided by phage titer for *E. coli* O157:H7 isolate.

**Table 2:** Survival of Shiga-toxigenic *Escherichia coli* (STEC) isolates on beef cattle hide pieces by the application of antimicrobial treatment and the multiplicity of infection of phages: STEC host.

STEC Isolate ^1.^	Sero-ID	Phages	Water	Control	Phage:Host MOI ^3^
USDA-FSIS 380-94	O157:H7	5.6 ± 0.2B ^2^	6.1 ± 0.2A	6.1 ± 0.3A	11.2
CDC 96-3258	O45:H2	5.8 ± 0.1AB	5.9 ± 0.1AB	5.8 ± 0.2AB	47.3
CDC 90-3128	O103:H2	5.2 ± 0.4C	6.0 ± 0.2A	5.9 ± 0.2AB	15.6
JB1-95	O111:H-	5.9 ± 0.1A	5.9 ± 0.2AB	6.0 ± 0.2A	41.7
CDC 97-3068	O121:H19	4.7 ± 0.3D	5.1 ± 0.1C	5.1 ± 0.2C	2.2
*Pooled Standard Error* = 0.07					

^1^ STEC isolates provided by J.B. Luchansky (USDA-Agricultural Research Service, Wyndmoor, PA, USA). ^2^ Values depict least squares means (log_10_ CFU/cm^2^) of STEC counts determined from analysis of variance. Values not sharing common letters (A,B,C,D) differ at *p* = 0.05 by Tukey’s Honestly Significant Differences (HSD) multiple comparisons test. Surviving STEC were enumerated on tryptic soy agar supplemented with 100.0 µg/mL rifampicin. Triplicate identical replicates were completed bearing three identically treated samples per replicate (*N* = 9). Antimicrobial treatments were: (1) STEC-inoculated non-treated group (Control); (2) STEC-inoculated, sterile distilled water (25 °C); and (3) STEC-inoculated with 10^8^ PFU/mL phage intervention-treated. ^3^ MOI: Phage multiplicity of infection (MOI) applied in the hide intervention study, calculated as the number of phages applied to hide surfaces divided by the number of bacteria retained on hide surfaces post-inoculation.
